# Supplementary material for: Hot spot prediction in protein-protein interactions by an ensemble system
Source: BMC Syst Biol. 2018 Dec 31;12(Suppl 9):132. doi: 10.1186/s12918-018-0665-8 (PMC6311905; doi:10.1186/s12918-018-0665-8)
Supplement: Supplementary file 2 — All relevant MATLAB code. A simple Matlab implement of our predictor. (ZIP 19,742 kb) [file 12918_2018_665_MOESM2_ESM.zip › Additional file 2/Read ASA/ASA website/SKEMPI/1F47A.htm]

 NetSurfP - Protein Surface Accessibility and Secondary Structure Predictions

  

|  |  |  |
| --- | --- | --- |
|  |  | NetSurfP - Protein Surface Accessibility and Secondary Structure Predictions  Technical University of Denmark |

  


---

```
# For publication of results, please cite:
# A generic method for assignment of reliability scores applied to solvent accessibility predictions.
# Bent Petersen, Thomas Nordahl Petersen, Pernille Andersen, Morten Nielsen and Claus Lundegaard
# BMC Structural Biology 2009, 9:51 doi:10.1186/1472-6807-9-51
#
# Column 1: Class assignment - B for buried or E for Exposed - Threshold: 25% exposure, but not based on RSA
# Column 2: Amino acid
# Column 3: Sequence name
# Column 4: Amino acid number
# Column 5: Relative Surface Accessibility - RSA
# Column 6: Absolute Surface Accessibility
# Column 7: Z-fit score for RSA prediction
# Column 8: Probability for Alpha-Helix
# Column 9: Probability for Beta-strand
# Column 10: Probability for Coil
E K  1F47A                  1    0.880 181.037   0.687   0.003   0.003   0.994
E E  1F47A                  2    0.668 116.665  -0.272   0.016   0.005   0.979
E P  1F47A                  3    0.388  55.100  -1.589   0.018   0.019   0.964
E D  1F47A                  4    0.583  84.010  -0.370   0.115   0.016   0.868
B Y  1F47A                  5    0.223  47.741  -0.572   0.184   0.043   0.773
B L  1F47A                  6    0.163  29.919   0.014   0.191   0.086   0.723
E D  1F47A                  7    0.468  67.468  -0.725   0.184   0.043   0.773
B I  1F47A                  8    0.069  12.839   0.719   0.622   0.015   0.363
B P  1F47A                  9    0.218  31.005  -0.018   0.717   0.014   0.269
B A  1F47A                 10    0.242  26.712  -0.950   0.879   0.010   0.111
B F  1F47A                 11    0.153  30.787  -0.635   0.879   0.010   0.111
B L  1F47A                 12    0.106  19.372  -0.237   0.879   0.010   0.111
E R  1F47A                 13    0.411  94.119   0.665   0.879   0.010   0.111
E K  1F47A                 14    0.576 118.565   0.173   0.717   0.014   0.269
E Q  1F47A                 15    0.521  93.051  -0.734   0.522   0.016   0.462
E A  1F47A                 16    0.415  45.755  -1.772   0.176   0.004   0.820
E D  1F47A                 17    0.948 136.607   0.067   0.003   0.003   0.994

Explain the output.  Go back.
```

---
